# Supplementary material for: Long range PCR-based deep sequencing for haplotype determination in mixed HCMV infections
Source: BMC Genomics. 2022 Jan 6;23:31. doi: 10.1186/s12864-021-08272-z (PMC8735729; doi:10.1186/s12864-021-08272-z)
Supplement: Supplementary file 2 — Additional file 2: Supplementary Figs. 1–3. Supplementary_Figures [file 12864_2021_8272_MOESM2_ESM.pptx]

## Slide 1
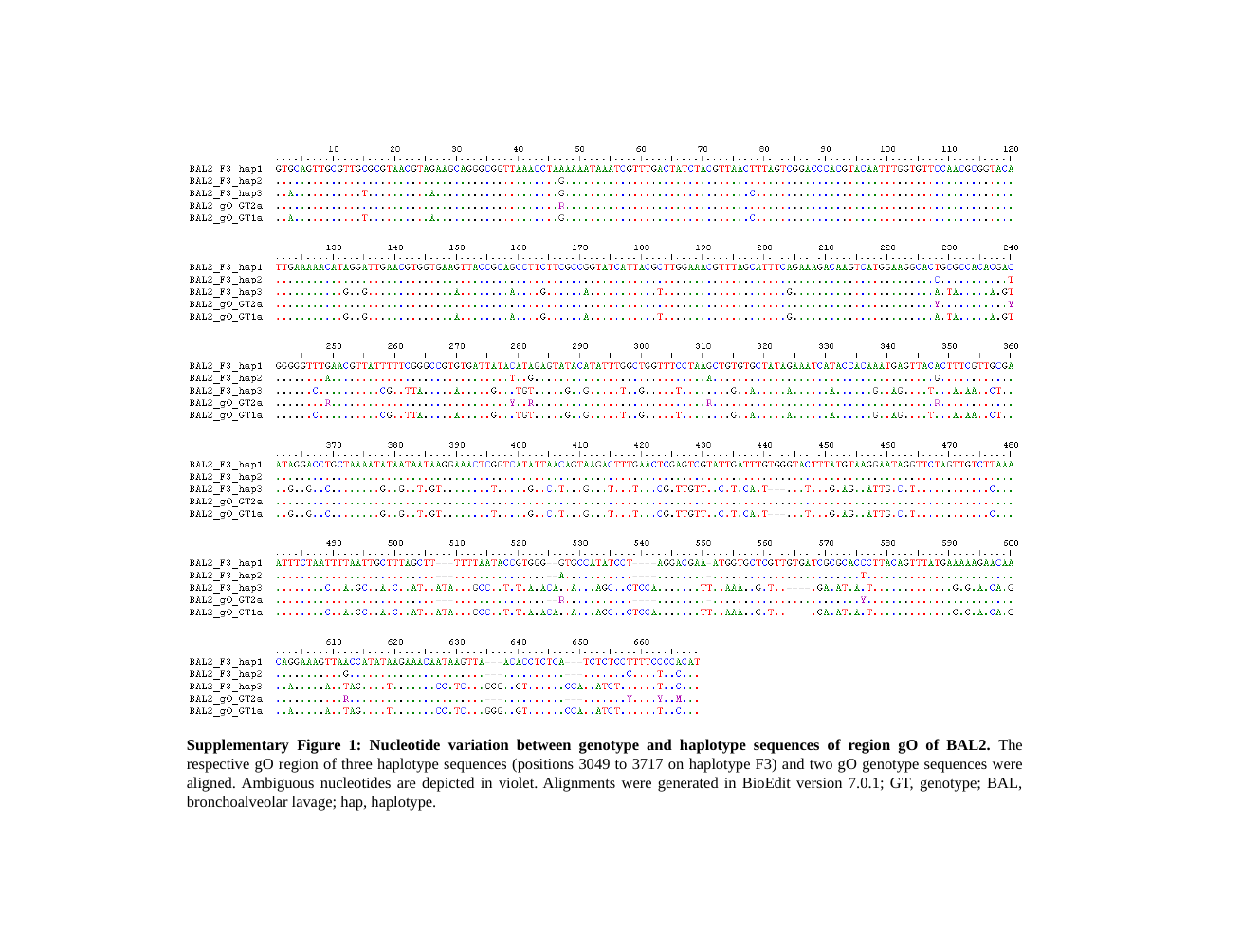

Supplementary Figure 1: Nucleotide variation between genotype and haplotype sequences of region gO of BAL2. The respective gO region of three haplotype sequences (positions 3049 to 3717 on haplotype F3) and two gO genotype sequences were aligned. Ambiguous nucleotides are depicted in violet. Alignments were generated in BioEdit version 7.0.1; GT, genotype; BAL, bronchoalveolar lavage; hap, haplotype.

## Slide 2
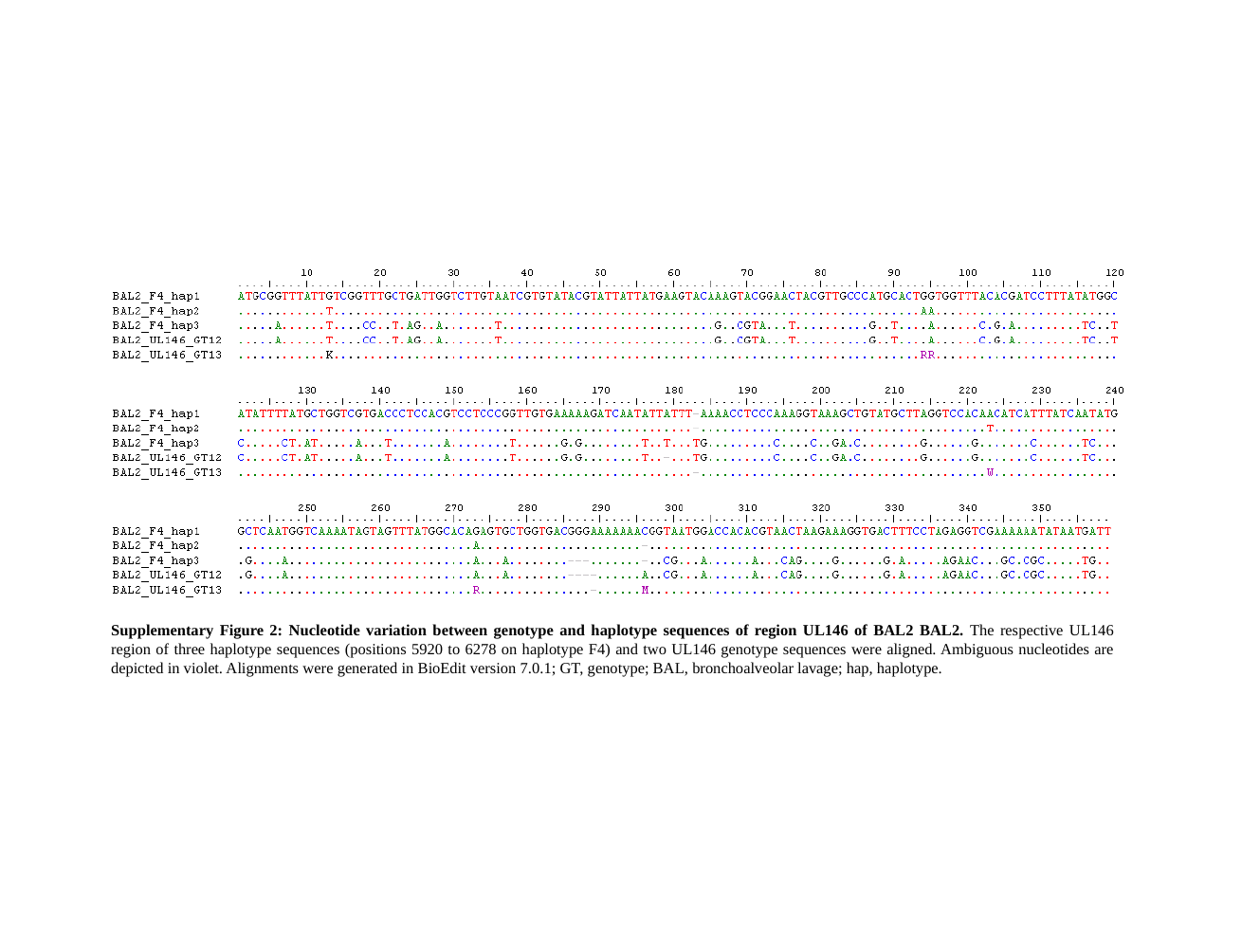

Supplementary Figure 2: Nucleotide variation between genotype and haplotype sequences of region UL146 of BAL2 BAL2. The respective UL146 region of three haplotype sequences (positions 5920 to 6278 on haplotype F4) and two UL146 genotype sequences were aligned. Ambiguous nucleotides are depicted in violet. Alignments were generated in BioEdit version 7.0.1; GT, genotype; BAL, bronchoalveolar lavage; hap, haplotype.

## Slide 3
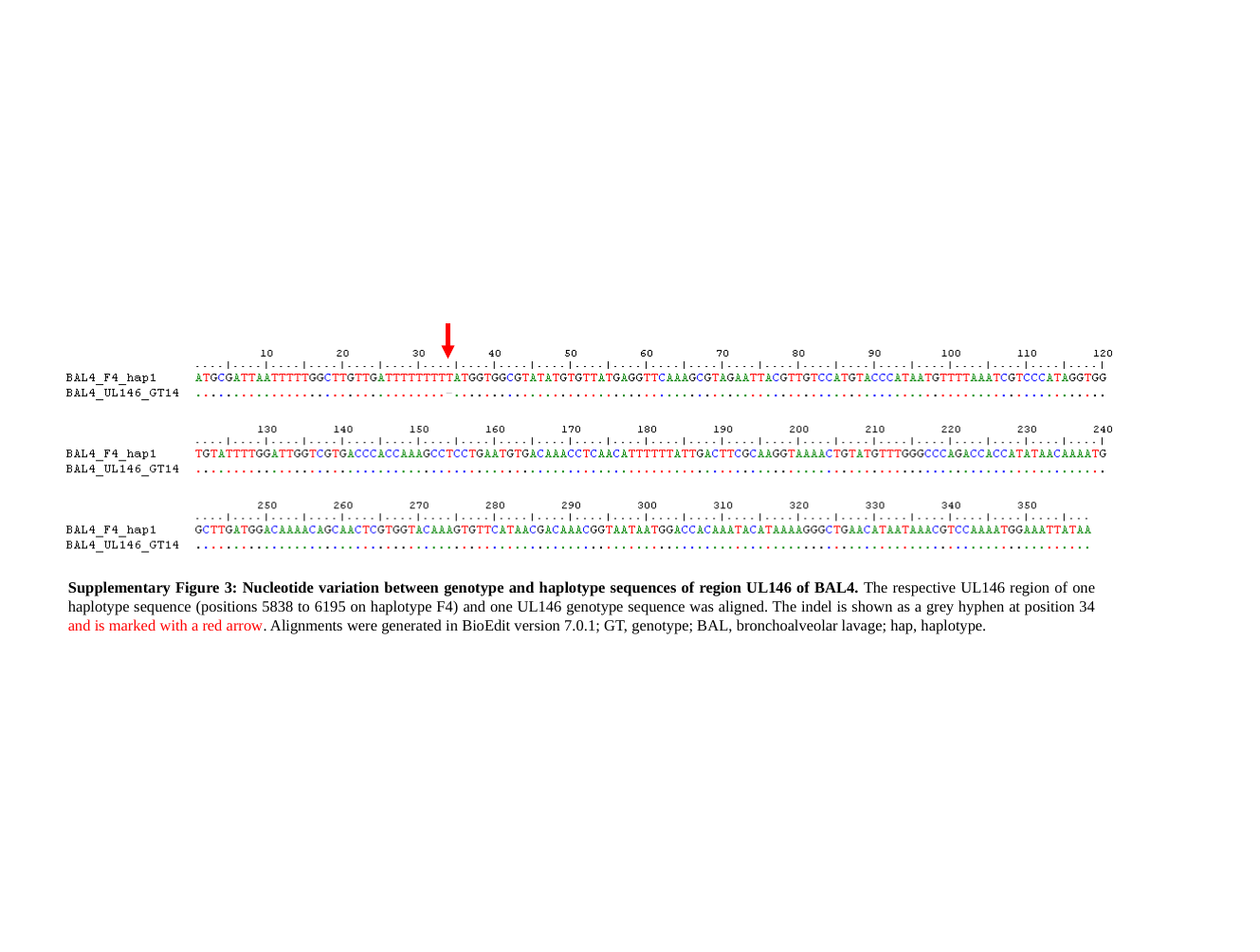

Supplementary Figure 3: Nucleotide variation between genotype and haplotype sequences of region UL146 of BAL4. The respective UL146 region of one haplotype sequence (positions 5838 to 6195 on haplotype F4) and one UL146 genotype sequence was aligned. The indel is shown as a grey hyphen at position 34 and is marked with a red arrow. Alignments were generated in BioEdit version 7.0.1; GT, genotype; BAL, bronchoalveolar lavage; hap, haplotype.
